# Opportunities for Managing Pain and Anxiety in the Intensive Care Unit Using Virtual Reality: Perspectives from Bedside Care Providers

**DOI:** 10.1089/jmxr.2023.0007

**Published:** 2024-03-19

**Authors:** Isabella P. Garito, Samantha Lewis-Fung, Brenna Lawson, Hannah Gabrielle Gray, Christopher Smith, Lora Appel

**Affiliations:** 1 School of Health Policy and Management, Faculty of Health, York University, Toronto, Canada.; 2 Michael Garron Hospital, Toronto, Canada.; 3 OpenLab, University Health Network, Toronto, Canada.; 4 Department of Psychology, Faculty of Health, York University, Toronto, Canada.

**Keywords:** virtual reality, intensive care unit, extubation, anxiety, pain, nonpharmacological

## Abstract

**Purpose:**

Virtual reality (VR) is a promising nonpharmacological tool to decrease pain and anxiety in the intensive care unit (ICU). Limited work, however, has been conducted on the functional implications of using VR to support intubated ICU patients. This study aimed to gather ICU providers' perspectives on implementing VR in the ICU to understand potential challenges and establish measurable outcomes.

**Materials and Methods:**

This was an exploratory qualitative study. ICU bedside care providers were shown how VR technology would look on an intubated mannequin, had the opportunity to experience VR, and took part in a semistructured interview.

**Results:**

Participants (*n* = 16) expressed an overall willingness to use VR in the ICU with intubated patients, indicating that VR may be particularly beneficial for prolonged intubation, but less optimal during extubation. Potential challenges included patient eligibility for use, informed consent, provider buy-in and workflow, and appropriateness of VR hardware/software.

**Conclusions:**

Successful adoption of VR in the ICU is contingent on provider involvement in designing staff training, best practices, and standard workflow for administering and evaluating the intervention. Although many concerns described by providers in our sample have potential solutions, staff preparation and family involvement remain critical to reducing the complexity of staff workflow for administering this intervention.

## Background

Intensive care units (ICUs) provide a critical role in treating patients with complex health conditions, including those with life-threatening illnesses. As a result, the management of patients' pain and anxiety present exceptional difficulties in the ICU.^1^ Patients in the ICU have a higher likelihood of experiencing anxiety due to the setting, including physical factors (medical devices, cables, etc.), unfamiliar individuals, invasive medical procedures, and loss of independence.^2^

Patients undergoing mechanical ventilation frequently experience pain due to the intubation process and ventilation itself, with discharged patients often able to recall the pain they felt in the ICU.^3^ Indeed, patients commonly experience physical and psychological symptoms of pain and anxiety during extubation,^4^ the process of removing an endotracheal tube (ETT) after mechanical ventilation has ended.^5^ Sedation and analgesics are most frequently used to manage pain and anxiety,^3,6^ as well as to prevent unintended extubations.^3^

Despite lighter sedation practices being used in the ICU since the early 2000s, oversedation continues to occur.^7^ Oversedation often leads to negative health outcomes, including delirium, a continued reliance on mechanical ventilation, memory loss, and in some cases, death.^3^ Furthermore, patients who are oversedated experience pain and delays in their ability to recuperate.^7^ Accordingly, there has been an uptake of interest in the use of nonpharmacological strategies to manage patients' pain and anxiety,^8^ including the use of music.^9^

Traditionally, virtual reality (VR) has found its niche in the entertainment, gaming,^10^ and simulation training space.^11^ Recently, research has increasingly focused on the affordances of its unique features for implementation in health care.^12^ In brief, VR-therapy is a nonpharmacological intervention often administered through a head-mounted display (HMD) to provide video and audio that completely immerses a user in the virtual environment.^13^

VR has successfully been used to manage pain and treat anxiety^12,14^ by temporarily distracting patients from distressing settings.^12^ This is achieved through obscuring/hiding the external environment and creating a sense of presence through multisensory immersion,^15^ resulting in a richer experience compared with nonimmersive technologies, such as a tablet with headphones. This tool has been used in various hospital care units, including emergency departments,^16^ general inpatient units,^17^ and during medical procedures.^18^

Use cases for VR in the ICU introduce new domains of scientific inquiry,^19^ and although more rigorous studies are still needed, early research suggests that VR has the potential to be effective in various ways.^19,20^ A recent scoping review investigating VR in the ICU concluded that many critical-care patients found the technology to be acceptable and enjoyable, although most of the 21 studies were in initial phases of assessing tolerability and clinical efficacy.^19^ Patients who used VR to increase relaxation saw an overall decrease in their anxiety and pain, among other improvements.^19^ Preliminary findings indicate that the risk of adverse events and/or cybersickness in ICU patients is low.^13,19^

To our knowledge, only two studies to date have examined the use of VR in intubated populations. The first was a feasibility trial conducted in 2021, which involved 21 providers (physicians, nurse practitioners, respiratory therapists, etc.) and 15 ICU patients, 6 of whom were mechanically ventilated.^13^ This study revealed that VR was well-tolerated and comfortable, with all patients reporting a sense of presence in the virtual environment.^13^ The second study similarly demonstrated the benefits of VR, successfully reducing anxiety in 10 intubated ICU patients.^21^

However, the application of VR during extubation remains unexplored. Although nonimmersive digital media strategies, such as music therapy, are currently utilized in ICUs to manage pain and anxiety more generally,^8^ their use during extubation is only beginning to be studied.^4^ Of note, in the mentioned studies, VR was administered by researchers rather than ICU staff, contributing to the lack of insight into provider perspectives on VR use. Often, end users, including health care staff, are overlooked when implementing new digital tools in care settings, leading to the common failure of digital interventions.^22^

To address this gap, it is crucial to involve health care staff throughout the design and development phases to optimize the success of implementation.^22^ With this objective in mind, we conducted an exploratory study to gather impressions and feedback from staff on the potential use and value of VR in the ICU. Our aim is to offer an enriched understanding of the potential effectiveness of VR as an intervention in intubated ICU patients from the perspective of those who will implement and administer it.

### Objectives

This study gathered the perspectives of ICU bedside staff on the use of VR to manage pain and anxiety in the ICU broadly, with a focus on extubation procedures. Specifically, we sought to explore the following:

1. The feasibility and appropriateness of VR, including staff willingness to use it.
2. Potential challenges, concerns, and barriers to the implementation of VR.
3. How VR compares with strategies currently being used for pain and anxiety in the ICU.
4. Potential metrics/assessments that would indicate VR's success in future studies.

## Materials and Methods

### Design

This was a qualitative exploratory study designed in conjunction with the ICU team at Michael Garron Hospital (Toronto, Canada). ICU bedside staff participated in 15-minute study sessions at a time and location in the hospital that was convenient for them to minimize the impact on workflow. To recognize their contribution, participants were provided with an honorarium in the form of a $5 coffee gift card.

### Setting

Data collection took place during a 2-week period in October 2022, at Michael Garron Hospital, a teaching hospital in Toronto, Canada. Ethics approval for this study was acquired by Michael Garron Hospital's Research Ethics Board on March 21, 2022 (reference number: 849-2110-Mis-366). Informed consent was obtained from all participants.

### Participants

A total of 16 ICU health care providers (10 registered nurses, 3 ICU physicians, 1 ICU resident, 1 respiratory therapist, and 1 personal support worker) were recruited to participate using a face-to-face approach. Purposive sampling occurred, with all health care staff involved in providing bedside care to patients in the ICU at Michael Garron Hospital eligible to participate in the study. Individuals with a pacemaker or history of epilepsy or seizures were excluded from this study to avoid contraindications as per VR device manufacturer recommendations. There were no participant dropouts.

### Methodology

Participation involved completing a one-on-one 15-minute session with a researcher, with no repeat or follow-up interviews. During each session, participants had the opportunity to (1) place a VR HMD on an intubated mannequin, (2) briefly explore VR environments themselves in the VR HMD, and (3) provide feedback through a semi-structured interview. Interviews were transcribed using paper-and-pen method. Transcripts were not provided to the participant for comment.

**FIG. 1.**
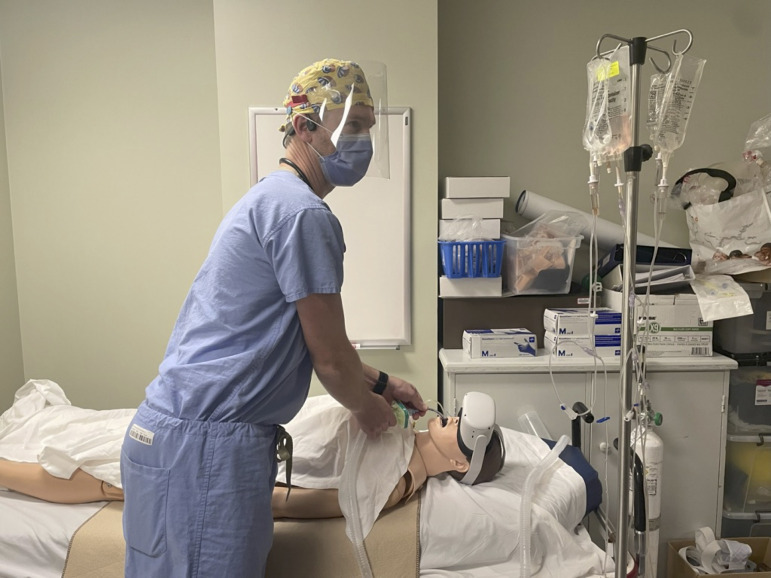
ICU simulation. The provider placed a VR head-mounted display onto a mannequin equipped with a triple lumen central line, ETT, ETT holder, and ETT filter and is preparing for extubation. ETT, endotracheal tube; VR, virtual reality.

Participants were first provided with a brief background on VR and presented with a scenario describing a health care provider using VR to manage pain and anxiety during an extubation procedure (Supplementary Appendix SA1). All participants were then asked to position a Meta Quest 2^23^ or Oculus Quest Go^24^ HMD onto a mannequin equipped with a triple lumen central line, ETT, ETT holder, and ETT filter (Fig. 1). The simulation was used as a way for participants to envision placing the HMD on an intubated ICU patient and identify potential challenges such as interference with workflow during an extubation.

**FIG. 2.**
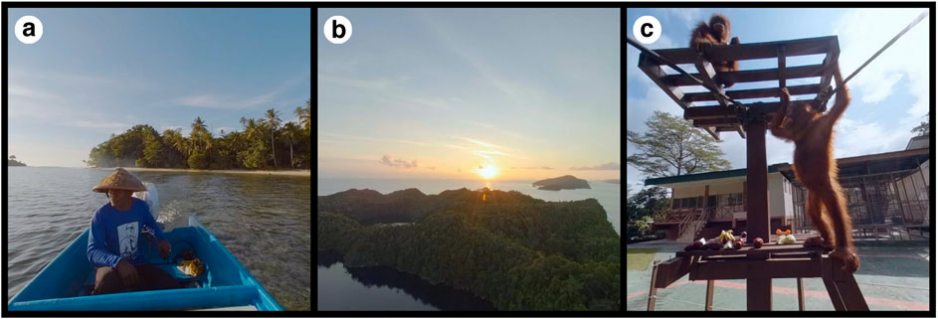
Examples of VR scenes shown to participants, screen-captured from the Ecosphere app.^23^ **(a)** Raja Ampat: Oceans, Indonesia; **(b)** Raja Ampat: From the air; **(c)** Borneo: Jungles, Malaysia.

After the simulation, participants were offered the opportunity to use the Meta Quest 2 HMD^23^ with two handheld controllers to watch nature-based 360° films from one of two free commercially available VR applications: Ecosphere (Fig. 2)^25^ or YouTube VR.^26^ All participants in this study opted to experience 360° VR content, with 14 watching videos from the Ecosphere app^25^ and 2 watching YouTube VR videos.^26^

Finally, participants took part in a semistructured interview that included the following five topics (Supplementary Appendix SA2):

1. Concerns with the characteristics of the VR headset for use with ICU patients.
2. Potential challenges with administering VR.
3. Measurable outcomes of successful VR.
4. Current strategies/interventions used in the ICU.
5. Other comments the participants wanted to share.

### Analysis

All records of feedback collected and open-ended observational notes underwent thematic analysis using the constant comparison method.^27^ Two authors (I.P.G. and B.L.) manually coded and sorted the data (e.g., Excel documents and paper-and-pen methods) over multiple iterations. The two raters met separately with each other and with the other research team members to discuss connections, ambiguities, and discrepancies within the data.

Themes that emerged from the study were reviewed by the PI and other team members at various stages of the analysis process to summarize the feasibility of using VR in the ICU and more specifically, during the process of extubation (Supplementary Appendix SA3). The Consolidated Criteria for Reporting Qualitative Studies (COREQ)^28^ checklist was used to ensure the validity of this qualitative study (Supplementary Appendix SA4).

Owing to unforeseen challenges, including a recent COVID-19 outbreak in the unit where demonstrations and interviews were conducted, coupled with funding constraints, the research team convened to assess the study's progress after the 16th interview. After thorough deliberations and preliminary analysis, the team determined that data saturation had been achieved, as no novel insights were emerging from the diverse group of health care professionals. Consequently, the decision was made to halt further data collection.

## Results

We first report on results shared by staff related to the feasibility of administering VR in the ICU, including overall impressions, perceived benefits, willingness to use the technology, and specific use cases (i.e., during extubation and otherwise). Next, we describe potential challenges that were raised and solutions suggested by staff to address these challenges, based on themes that emerged from our analysis. Finally, we report on participants' descriptions of other interventions currently being used in the ICU to manage pain and anxiety and measures that would indicate therapeutic success of VR in this patient population (see Supplementary Appendix SA5 for complete results).

### Feasibility

Most participants (11/16, 68.8%) were overall positive about introducing VR in the ICU, indicating ways that it may be beneficial, including for intubated patients. The remainder were either more mixed/unclear (3/16, 18.8%) or negative (2/16, 12.5%) when describing their impressions of its feasibility and potential benefits. Regardless of outlook, all participants (16/16, 100%) foresaw challenges with administering VR and described cases where VR would not be appropriate in the ICU, which are discussed in further detail in the following section.

The ideal ICU candidate for VR therapy was described by participants in various ways including being awake, calm, able to consent, at least somewhat alert, able to tolerate the headset weight, younger in age, and not delirious. ICU use cases—areas where VR could be used—included entertaining patients who are bored (in particular, intubated patients with an extended length of stay), alleviating anxiety and pain during sedation reduction, after extubation, and during procedures known to elicit anxiety such as wound care.

Several participants, including individuals who described more hesitancy toward using VR in the ICU, suggested the potential therapeutic benefits of VR in other areas of the hospital, including colonoscopy, endoscopy, IV insertions, dialysis, palliative care, dentistry, and birthing units. Indeed, many (6/16, 37.5%) participants volunteered that they felt relaxed and calm after trying VR during the study, for example, stating, *“I felt calm. I felt like my anxiety had gone down and I relaxed”* (participant 1), *“How long can I stay in this [VR] for?”* (participant 6) and *“They should have this [VR] for the workers!”* (participant 16). One participant jokingly asked if they could return after their shift to use the HMD and relax again.

Regarding the feasibility of using VR during extubation, most participants either felt that VR would not be helpful for reducing anxiety or speeding up the extubation process (6/16, 37.5%), or were undecided (7/16, 43.8%). However, one participant described how extubation may be an ideal time to use VR because sedation is decreased and patients are required to be alert, a situation during which VR may serve as a potential stimulating distraction. Only one participant (1/16, 6.5%) suggested that the HMD might directly interfere with the procedure (i.e., that the headset's weight might make it difficult for patients to maintain the necessary upright head position).

The most commonly cited concern was that incorporating a VR HMD would result in increased preparation time for what is usually a quick procedure. In addition, there were concerns the HMD may pose a safety risk by making it more difficult for patients to hear and follow instructions, causing unsafe head movements (e.g., turning their head toward sounds within the virtual scene), feelings of panic from the forceful coughing that typically occurs after extubation, or obstruction if an emergency re-intubation is necessary. As participant 1 described, *“I think it would be more beneficial for already intubated patients that are bored and have nothing to look at.”*

### Challenges

A number of challenges were raised by participants when considering the use of the VR technology in this specialized environment. These challenges fell under the following themes, which we discuss in further detail hereunder: (1) Patient, (2) Provider, and (3) Technology.

#### Patient

##### Eligibility for use

There was consensus among participants that VR would not be appropriate for use within the entire ICU patient population, necessitating criteria outlining eligibility for use and contraindications. Two primary concerns were described: first, that many patients in the ICU have a reduced level of consciousness and consequently, would not benefit from an altered reality; and second, the possibility of the intervention increasing anxiety, agitation, or confusion in patients.

One participant mentioned that since the majority of patients are older, confusion is especially a concern. However, as another participant explained, determining eligibility may be difficult: *“It isn't easy to know or predict how someone is going to react”* (participant 10). Participants' suggestions of criteria related to behavior/state and diagnosis/injury that would indicate unsuitability for using VR are summarized in Table 1.

**Table 1.** Criteria Suggested by Participants (*n* = 16) to Indicate Potential Ineligibility for Virtual Reality Use

	Participants who mentioned the criterion, ***n*** (%)
Behavior/state	
Confused	4 (25)
Delirious	3 (18.75)
Agitated	2 (12.5)
Drowsy	1 (6.25)
Combative	1 (6.25)
Diagnosis/injury	
Neck collar/stiff neck/spinal cord injury	2 (12.5)
Tinnitus/Sinusitis	1 (6.25)
Meningitis	1 (6.25)
Dementia	1 (6.25)
Vision deficits	1 (6.25)
Post-flap/softer skull	1 (6.25)

##### Gaining consent

Staff emphasized the importance of patients being sufficiently alert and aware to provide consent before using VR and during (i.e., the ability to purposefully gesture or express their like or dislike for the therapy). Gaining consent from intubated patients was described as a significant potential challenge, particularly in cases where language barriers are present. Some participants suggested that it might be feasible to explain the therapy to patients who are awake and alert enough to respond to yes/no questions.

Participants also suggested weaning the patient off sedation to enhance alertness to gain consent. Two participants (2/16, 12.5%) highlighted the involvement of families in the consent process, with one participant emphasizing that decisions regarding consent are often made by the family on behalf of their incapacitated loved ones.

#### Provider

##### Impact on workflow

Participants raised a number of questions and concerns related to staff roles and processes for administering VR therapy and managing the VR equipment. Infection Prevention and Control (IPAC) of the HMD was the greatest concern described (8/16, 50%), especially for extubation where aerosols are present. At the same time, some participants recognized that there may be easily implementable solutions, such as having a cover for the HMD (recommended by two participants), whereas another mentioned that equipment is often shared between patients and that, “*there just has to be a protocol for cleaning*” (participant 10).

Establishing role clarity and administering appropriate training will be essential. Indeed, questions arose about which staff would know how to use the technology (6/16, 37.5%), ensure that the HMD is set up and ready before any procedures (3/16, 18.8%), and the potential difficulty of placing the HMD on the patient's head (2/16, 12.5%).

It was also suggested that an additional staff member would be required to administer VR therapy, impacting staff resourcing: *“The first person would hold the head as [the patient's head] may be heavy and the second person would place the headset on the patient”* (participant 5). Clear guidelines for handling, storing, and maintaining the equipment would also be necessary (*“[what if it gets] lost or damaged and needs repair?”* (participant 16)).

##### Buy-in

Participants emphasized the need for staff to see evidence of the value of VR therapy to be willing to implement it and feel comfortable using it with patients. One participant noted that older staff members may not be as comfortable using new technology, which may pose a challenge in the success of VR implementation. A potential solution suggested was to conduct an assessment of staff readiness to accept VR therapy.

#### Technology

##### Hardware

The weight of the HMD was the most common concern described with respect to the hardware (6/16, 37.5%), with one participant suggesting that, *“This could only be approved if a lighter headset was available”* (participant 3). There were also concerns regarding the fit of the HMD (4/16, 25%) including tightness around the eyes and trouble achieving a comfortable fit, specifically the gap between the user's face and the HMD impacting the viewing experience. Other concerns included the cost/durability of the HMD/charging cables, and patients not being able to use the hand controllers, which may impact the patient's ability to move around in the virtual environment.

##### VR software and content

There was apprehension (5/16, 31.25%) regarding the staff's inability to know if certain images or sounds would elicit fear in patients, which in turn could result in unpleasant experiences and increased anxiety. For example, while using the VR HMD, one participant mentioned *“This video has water, but what if the patient has a fear of water?”* (participant 14). As explained by this participant, staff may not know ahead of time about patient preferences or fears.

Another participant noted that when a patient's bed is raised, the patient sometimes experiences a sense of falling and explained that VR has the potential to elicit a similar response, which may provoke anxiety and/or discomfort in patients. Two participants experienced mild anxiety after watching videos that began with aerial views and although both indicated that VR would be beneficial for decreasing anxiety in patients, they agreed users must be presented with more appropriate scenes. Other concerns described were the risk of experiencing motion sickness, and the inadequate image resolution of some of the content.

### Interventions currently used in the ICU to manage pain and anxiety

To understand the potential impact of VR in the ICU, we asked participants to describe existing standard-of-care strategies for pain and anxiety. Their responses are summarized in Table 2.

**Table 2.** Current Therapies and Interventions Being Used for Patients in the Intensive Care Unit

Participants who mentioned each strategy ***n*** (%)			
Strategies	Entertaining/comforting intubated patients (***n*** = 12)	Successfully extubating (***n*** = 14)	Reducing pain and anxiety (***n*** = 15)
Family/provider interaction			
Reassurance (e.g., *talking to patient and hand holding*)	6 (50)	3 (21.4)	4 (26.66)
Explaining procedure to patient	—	7 (50)	—
Reposition, sit with, and/or distract patient	—	—	2 (13.3)
Family member presence/touch/bring belongings	2 (16.6)	1 (7.14)	1 (6.66)
Medical			
Medication	2 (16.6)	1 (7.14)	9 (60)
Procedural (e.g., *quick procedure, ensure everything is procedurally ok, and remove secretions quickly*)	—	4 (28.57)	—
Digital media			
YouTube, music, movies, TV, and computer	6 (50)	—	5 (33.33)
Environmental			
Reduce stimuli and crowding of room	—	—	2 (13.3)

Reassurance and family member presence were the most consistently described strategies for a variety of scenarios. Medications were described as most effective for managing anxiety and pain specifically, and procedural strategies (removing secretions quickly, etc.) were unique to ensuring successful extubation. Although digital media (e.g., YouTube, movies, and music) was used both to entertain and distract intubated patients from the ICU environment and manage pain and anxiety, it was not mentioned as a strategy for ensuring successful extubation.

Two key challenges with current strategies were described. First, participants reported that it can be difficult to know if these interventions are helpful for managing pain, anxiety, or boredom for a given patient. As one participant said, *“Patients' families bring in belongings, play music, have photos. [But I am] unsure if it does anything”* (participant 10). Another explained how, *“Some nurses put on YouTube videos for patients in the ICU. I don't know [if it is helpful because the patients] don't have much of a choice.”* (participant 6).

Second, the ability to select appropriate content for digital interventions was described as *“depending largely on the family”* (participant 10), whose presence may vary. Combined, these challenges have resulted in available options falling into disuse. One participant, however, volunteered that VR may have an advantage in this regard over nonimmersive media: *“There is a TV just collecting dust in the ICU—but I could see how [VR] would be more immersive”* (participant 10).

### Measures of success

Patient anxiety/distress, pain, irritability, relaxation, and experience/satisfaction, as well as VR-specific outcomes (i.e., comfort and engagement while in VR, motivation to use VR) were listed by participants as measures that would indicate the success of VR in the ICU. In addition, provider factors, including satisfaction, and accessibility of the intervention were described as important to measure. Overall, participants emphasized the importance of using mixed methods (subjective and objective measures) and supportive communication strategies in considering current challenges inherent to communicating with acutely ill, sedated, and intubated ICU patients.

#### Subjective measures

Participants suggested using both subjective observational (5/16, 31.25%) and patient self-report (11/16, 68.75%) tools to evaluate the impact of the intervention on patient outcomes. Specific observational strategies described included observing patient body language, gestures, and movements for signs of distress/relaxation while in VR and using the Critical Care Pain Observation Tool, which is currently employed to measure pain when patients are unable to communicate.^29^

Suggested self-report measures included validated scales (e.g., scales assessing anxiety/post-traumatic stress disorder), experience ratings, and satisfaction surveys, as well as interviewing the patient. Specific strategies to support communication suggested by participants included using questions that require a yes/no answer, visual supports (e.g., happy face scales), and establishing gestures the patient can use if they want VR off/do not like it.

#### Objective measures

Several participants (9/16, 56.25%) also suggested objective physiological/medical measures that could be used as quantitative data to conclude the success of VR in patient use. Participants recommended tracking vital signs (blood pressure and heart rate), and biometric monitoring pre- and post-VR use. Tracking sedation and antipsychotic use is another potential tool, measuring success by assessing whether those who received VR therapy were administered less medication. For extubation, data on the number of successful extubations, reintubation rates, and the time required to complete the procedure should be gathered.

## Discussion

The study compiled perspectives from diverse stakeholders on the feasibility of introducing VR therapy to patient care in the ICU. Overall, VR appears to be feasible in the ICU for intubated patients, particularly for prolonged intubation and pre-/post-extubation, but not during extubation procedures due to time constraints and safety concerns. Of note, participants suggested alternative uses for VR therapy across clinical settings, demonstrating the potential value they saw in VR being able to reduce pain and anxiety, and their willingness to use the intervention.

**FIG. 3.**
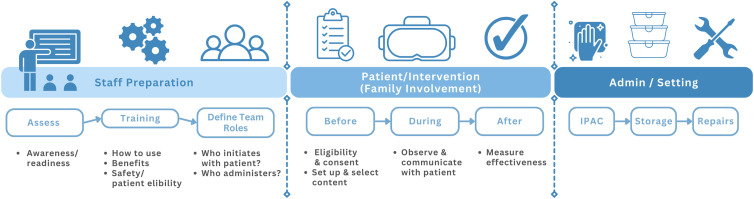
Timeline for implementing VR in the intensive care unit: implementation processes and potential challenges.

Nevertheless, despite an overall positive sentiment toward using VR in the ICU, all providers in this study identified potential challenges that could impact the feasibility of both the implementation and evaluation processes. These challenges were associated with provider workload, patient reception, and factors related to the technology. Figure 3 illustrates these challenges on a timeline, outlining key activities and considerations for implementing VR in the ICU.

Our findings suggest that ICU staff require training on the benefits of VR, as well as information on how to safely deploy it, including how to develop standard work processes for administering the intervention. Participants indicated implementing VR in the ICU may be met with resistance by clinicians and family members alike due to unfamiliarity with the technology, doubts that can be assuaged by ensuring alignment with institutional goals and comprehensive planning to empower staff.

Our findings echo other studies that have emphasized the importance of providing adequate and effective staff training to promote provider buy-in. For instance, initial provider resistance was also observed in a previous study where providers administered VR to veterans with dementia for the purpose of managing behaviors related to acute distress.^30^ Providers' perspectives changed, however, as they learned about the technology and became more comfortable using VR to manage patients' behaviours.^30^

Some participants in this study noted that longer training sessions and visual tools would have been beneficial.^30^ Other studies have suggested training through role-play activities may be particularly helpful for practitioners to develop the necessary skills^31^ and confidence to administer the intervention.^32^ Likewise, online modules are found to be effective, with the potential to include in-person training and mentorship.^33^

Along with equipment training and personnel resources, our findings also suggest that it will be critical to establish and train staff on a clear set of ICU-specific guidelines outlining indications for VR use, as well as guidelines for obtaining informed consent, and strategies for administering and measuring the impact of VR therapy. As highlighted by participants in our sample, ICU patients present with varied and fluctuating cognitive statuses and abilities to communicate, as well as physical limitations that may impact the intervention's safety and efficacy. Along these lines, our findings also illustrate several VR hardware, software, and content considerations unique to designing VR therapy to be used in this setting.

When establishing indications for VR use, it will be important to rely on research, in addition to providers' opinions, to ensure that special populations are not excluded from therapy without evidence/cause. Most criteria suggested by participants in our sample have intuitive merit at face value; for instance, that ICU patients would need to be *“awake and alert and not baseline confused”* (participant 9) to safely benefit from VR is a logical assumption.

Still, one provider mentioned that a diagnosis of dementia should be an exclusion criteria and others expressed hesitancy with regard to using VR with older patients in general, when in fact the therapeutic use of VR with older adults, including people living with dementia, is well-documented.^34,35^ Early research has also indicated that VR can be effectively used to treat patients with other exclusionary criteria suggested by participants, including spinal cord injuries^36^ and vision deficits.^37^

Pre-existing strategies and processes can be leveraged to address challenges related to communication and cognitive status. For example, informed consent might be obtained in the preoperative phase, such as the preanesthetic consultation, especially when ICU admission is expected. Continuous consent monitoring and supportive communication strategies such as yes/no questions and body language observation, as well as involving patients' chosen next of kin, can also enhance the consent process. Since providers may not always be able to receive feedback from patients, it is crucial to find alternative methods of communication to address video preferences, safety concerns, and outcome measures.

This could include having family members present at the time of the VR session^32^ as they should be knowledgeable about the patient's likes/dislikes,^38^ providing a button to press to request assistance with removing the HMD^32^ or establishing hand gestures to communicate preferences.^39^ Future studies on VR use for intubated patients should use a combination of measures to determine the success of VR therapy in this population, including observational tools, such as ObsRVR, originally designed to measure reactions to VR in the dementia population.^40^

Given the anticipated challenges of VR use in the ICU, the VR system should be modified and appropriate content should be selected in order for patients to experience benefits, while reducing setup complexities for staff and patients. The VR demonstration in this study involved two readily available applications that included some scenes with first-person motion and required initial setup inside the HMD before putting the HMD on the patient's head. From this arose apprehensions regarding motion sickness and added workload for providers.

Although there was no reported simulator sickness or interference with the intubated patient's ETT in two studies involving intubated patients,^13,21^ the limitations of existing applications point to the need for a customized companion application that uses a secondary device (i.e., a tablet or computer). Use of a companion application would facilitate selection and monitoring of VR content and reduce reliance on HMD controllers.^41^ Such an application could ideally provide access to carefully vetted content, including VR selections that are relaxing, generalizable to a diverse range of preferences, and have limited first-person motion to reduce the possibility of simulator sickness.^42^

In addition, participants expressed concern that ICU patients may be lying down and unable to turn their heads, making it difficult to see the intended field of view in VR, and also letting in light through the gap between the patient's nose bridge and the HMD's facial interface. These same concerns were identified in a recent case report describing VR's use while reclined in a dental chair.^43^

Possible work-arounds include using two-dimensional YouTube videos that can be adjusted to a higher or lower position in the HMD,^43^ and placing a soft material over the patient's nose bridge to block outside light, should the patient find this to be a distraction. Separate accessories to limit light flow into the HMD may be available for purchase for newer HMD models.^44^

Finally, hospitals will need to create standard procedures for proper cleaning, storage, and repair of VR equipment, in conjunction with ICU staff. IPAC is essential given that bacteria can grow on high contact areas within the ICU^45^; for example, the HMD could be exposed to aerosols during certain medical procedures, including extubation. However, as noted by one participant, the involvement of the IPAC teams and the development of cleaning protocols (similar to those introduced for other mobile devices, e.g., smartphones) could make the sharing of a VR headset possible between patients.

CleanBox Technology™^46^ has been used sanitize the HMD^47,48^ in previous studies to ensure cleanliness, as it uses UV rays to disinfect once the HMD and controllers have already been cleaned with disinfecting wipes.^47^ After proper sanitation, providers should be aware of standard procedures, including ensuring the HMDs are charged for their next use, placing them in their associated boxes to avoid damage, and storing them in an accessible location within the unit.

The exponential pace of technological development^49^ suggests that innovative tools such as VR are poised to integrate into the ICU, and hospitals should proactively prepare for the introduction of these potentially transformative interventions in this rapidly evolving field. Two previous studies found that VR was feasible to use with intubated ICU patients when administered by a researcher;^13,21^ however, given the small sample sizes and lack of separate cohorts, the intervention's effectiveness warrants further investigation, under real-world conditions.

Our next step in this area of research is to gauge the interest of ICU managers and staff regarding implementing VR programming in the scenarios suggested by participants. The VR intervention would be administered by the ICU practitioners and include staff involvement in all areas of the programming development (e.g., training, intervention design, implementation, and evaluation).

### Strengths and limitations

A notable strength of this study was the use of participatory design methodology; we were able to identify only one other study that gathered feedback from ICU practitioners on the use of VR.^19^ Integrating health care practitioners through participatory design research, particularly in the development and design of new technologies, facilitates identification of potential challenges and concerns before implementation.^50^ This inclusion of end users can highlight unmet needs, pain points, and reduce the likelihood of unanticipated errors, thereby supporting a smoother implementation in health care settings.^50^

Some limitations to this study's design must be taken into account when considering the generalizability of our findings. Although the mannequin used for demonstrations was fitted with the same equipment used for intubated patients requiring mechanical ventilation, it does not exactly replicate the clinical scenario and could result in unanticipated challenges not foreseen by participants.

Future studies should delve into providers' perspectives when administering VR in the ICU with real patients. Finally, the absence of personal health information collection, such as sex and/or gender, and reliance on a small sample size of health care providers from a single hospital in a large Canadian urban center, may restrict the comprehensive reflection of ICU staff more broadly.

## Conclusion

This exploratory study provides insight into the potential use cases and challenges associated with implementing VR in the ICU to reduce pain and anxiety in intubated patients from the perspective of bedside ICU health care providers. Overall, providers demonstrated willingness to use VR for this purpose and many expressed optimism about its potential benefits. Our findings both support the feasibility of using VR with intubated patients and highlight the critical role of bedside care staff in supporting the implementation of this intervention.

Although many of the concerns raised by participants have known solutions (e.g., IPAC and appropriate video content), additional work will be required for VR to be successfully adopted in ICUs and for providers to feel confident in using the technology. Specifically, it will be essential for providers to receive adequate training on how to use the technology and for clear procedures for its use to be established, including selecting appropriate patients, gaining consent, and measuring impacts of the VR intervention. For VR therapy in the ICU to gain traction, input through participatory design as research continues will be key to overcoming roadblocks for both health care staff and patients.

## Ethics Approval and Consent to Participate

Ethics approval for this study was acquired by Michael Garron Hospital's Research Ethics Board on March 21, 2022. Informed consent was obtained by all participants before their enrollment in the study.

## Consent for Publication

Informed consent for publication was obtained from any individuals shown in photos.

## Availability of Data and Materials

The data sets supporting the conclusions of this article are included within the article and its additional files.

## Supplementary Material

Supplementary Appendix SA1

Supplementary Appendix SA2

Supplementary Appendix SA3

Supplementary Appendix SA4

Supplementary Appendix SA5

## Abbreviations Used

ETT: endotracheal tube
HMD: head-mounted display
ICU: intensive care unit
IPAC: infection prevention and control
VR: virtual reality
